# Influence of temporal delay in SWEEPS dual-pulse Er: YAG laser-activated irrigation on fluid dynamics in confined domain environment: an in vitro study

**DOI:** 10.1007/s10103-026-04947-9

**Published:** 2026-07-22

**Authors:** Xinyu He, Kedi Jihu, Yue Yu, Xin Hu, Chong Pan, Jizhi Zhao

**Affiliations:** 1 Department of Stomatology, Peking Union Medical College Hospital, Chinese Academy of Medical Sciences and Peking Union Medical College, Beijing, 100730 China; 2 The First Hospital of Tsinghua University, School of Clinical Medicine, Tsinghua Medicine, Tsinghua University, Beijing, 100016 China; 3 Fluid Mechanics Key Laboratory of Education Ministry, Beihang University, Beijing, 100191 China

**Keywords:** Er:YAG laser, Laser-activated irrigation, SWEEPS, Dual-pulse, Root canal therapy, Fluid dynamics

## Abstract

The shock wave-enhanced emission photoacoustic streaming (SWEEPS) technique delivers synchronized dual-pulse Er: YAG laser energy to enhance fluid dynamics during laser-activated irrigation. The temporal delay (Tp) between consecutive pulses is a key parameter, but its influence on fluid dynamics within a confined root canal environment remains unclear. This in vitro study investigated the effects of different Tp settings on bubble morphology and transient apical peak pressure (TAPP) in a simulated root canal model. A transparent single-rooted resin anterior tooth model (apical diameter: 0.30 mm, taper: 6%, root canal length: 12 mm) was fabricated. The root canal was filled with deionized water. SWEEPS dual-pulse Er: YAG laser irrigation (20 mJ/pulse, 20 Hz) was performed at five Tp settings: 120, 210, 300, 400, and 500 µs. Bubble dynamics within the root canal were recorded using a high-speed camera system (200,000 fps). The grayscale integral of bubble images was calculated using MATLAB as a surrogate for relative bubble volume. Simultaneously, TAPP at the root apex was measured using a high-frequency pressure sensor (50 kHz). Four repeated measurements were performed per Tp group. Data were analyzed using one-way ANOVA followed by Tukey’s HSD post-hoc test (α = 0.05). Both bubble morphology and TAPP varied significantly with Tp. At Tp = 120 µs, the bubbles generated by the first and second pulses coalesced into a single larger bubble, whereas at Tp = 300–500 µs, two independent bubbles formed sequentially. The grayscale integral (secondary cavitation volume surrogate) was significantly higher at Tp = 120 µs ((0.074 ± 0.010) ×10^6^ pixel) than at 210 µs ((0.052 ± 0.012) ×10^6^ pixel), P < 0.05), and significantly higher at 500 µs ((2.767 ± 0.195) ×10^6^ pixel) than at 300 µs ((1.926 ± 0.06) ×10^6^ pixel) and 400 µs ((1.986 ± 0.135) ×10^6^ pixel, both P < 0.05). TAPP followed a similar pattern: 120 µs produced significantly higher apical pressure than 210 µs (3.520 ± 0.291 vs. 2.835 ± 0.047 kPa, P < 0.05), and 500 µs produced significantly higher pressure than 300 and 400 µs (3.398 ± 0.068 vs. 2.807 ± 0.099 and 3.128 ± 0.182 kPa, both P < 0.05). No significant differences in TAPP were observed between 120 and 500 µs (P > 0.05). In this in vitro resin root canal model with deionized water, SWEEPS dual-pulse Er: YAG laser-activated irrigation with Tp = 120 or 500 µs produced greater secondary cavitation volume and higher apical pressure than intermediate Tp values (210–400 µs), indicating that Tp significantly influences fluid dynamics under the tested experimental conditions.

**Supplementary Information:**

The online version contains supplementary material available at 10.1007/s10103-026-04947-9.

## Introduction

Root canal therapy is the preferred treatment method for pulp and periapical diseases, and the key to its therapeutic effect lies in the effective control of infections within the root canal system [1, 2]. The final irrigation technique is a key step in root canal therapy and includes methods such as needle-based, ultrasonic, and laser-activated irrigation [3–6]. In recent years, many studies have shown that the SWEEPS technique based on Er: YAG lasers has the potential to achieve better infection control [2, 7–9]. The effect of the SWEEPS technique lies in controlling the Tp between the two consecutive laser pulses to regulate the physical interaction between the two laser-induced bubbles. Ultimately, different physical interactions result in different irrigation effects [10–12]. Yang et al. [9] found that SWEEPS can emit synchronous laser pulses to accelerate the bursting of vapor bubbles and enhance fluid movement and transient shear stress within the root canal, thereby cleaning the main root canal and irregular areas within the root canal, and effectively improving the flushing effect at deeper positions within the root canal. Multiple studies have shown that SWEEPS has better potential for removing debris from root canals than photon-induced photoacoustic streaming (PIPS), acoustic waves, and ultrasound-activated irrigation [8, 9, 11, 13].

The factors that affect the cleaning efficacy of SWEEPS technology include pulse energy, Tp, and dental anatomical structure. Tp refers to the time delay between two consecutive Er: YAG laser pulse energy inputs, which is a key parameter in SWEEPS technology. Its duration determines the fluid-dynamic intensity of the interactions between the two bubbles [14]. Studies have shown that changing the Tp setting in free water can change the interaction between two vapor bubbles, which may enhance the clinical application of laser-activated irrigation [15, 16]. Previous studies have investigated the role of SWEEPS in free water bubble dynamics and identified three Tp dependent interaction mechanisms (coalescence, collision, and separation), with the strongest bubble collision occurring at Tp = 360–440 µs [16]. However, bubble dynamics in the restricted root canal differ significantly from free water. So far, there is no systematic study describing how Tp affects fluid dynamics within root canal models,. prompting the current investigation.

Consequently, from a clinical perspective, the lack of such data limits the ability of dental practitioners to select optimal laser parameters for root canal disinfection. Identifying the most effective Tp setting could potentially improve debris and smear layer removal, and enhance the long-term success rate of endodontic therapy, particularly in anatomically complex root canal systems.

Therefore, the aim of this study was to investigate the effect of the SWEEPS technique on the fluid dynamics of Er: YAG laser-activated irrigation under different laser Tp values. High-speed photography was used to record the dynamic process of vapor bubbles under different Tp conditions, and this was combined with the TAPP measurements to systematically analyze its influence on the fluid-dynamic effects of Er: YAG laser activation and irrigation. Thus, this study aimed to provide theoretical support for the principles of fluid mechanics in SWEEPS and basic empirical evidence for its clinical promotion and application.

Accrodingly, the null hypothesis tested in this study was: There is no significant difference in secondary cavitation volume (assessed by grayscale integral) or TAPP among different Tp settings (120, 210, 300, 400, and 500 µs) during SWEEPS laser-activated irrigation in an in vitro resin root canal model filled with deionized water. And the alternative hypothesis was: Different Tp settings produce significantly different fluid dynamics.

## Materials and methods

### Preparation of resin model for single anterior tooth tube

**Fig. 1:**
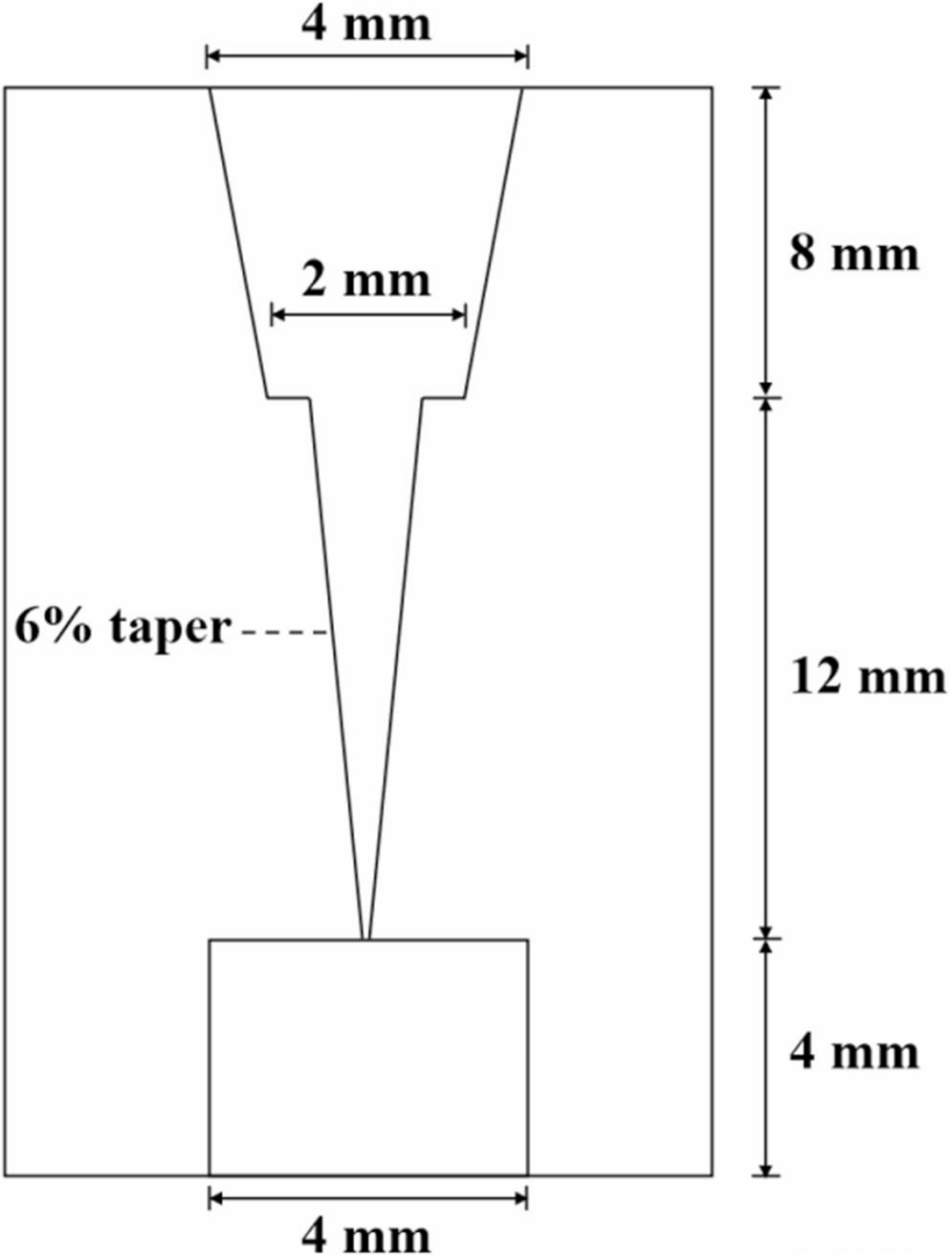
Schematic diagram of single-rooted resin model of anterior teeth

A resin model (NanoArch S140, Chongqing Mofang Precision Technology Co., Ltd., China) of a single-rooted anterior tooth canal was used, with a root apical foramen diameter of 0.30 mm, a taper of 6%, a pulp chamber height of 8 mm, and a root canal length of 12 mm. The diameters of the pulp chamber top and bottom were 4 mm and 2 mm, respectively (with a measurement accuracy of 1 μm under a microscope). The cylindrical base connecting the root apex and the pressure data acquisition card had a height and diameter of 4 mm, as shown in Fig. 1. Three independent resin models were fabricated under identical conditions. Each Tp condition was tested on all three models, with four repeated measurements. The results from the three models were combined for statistical analysis.

### Experimental working mode

**Fig. 2:**
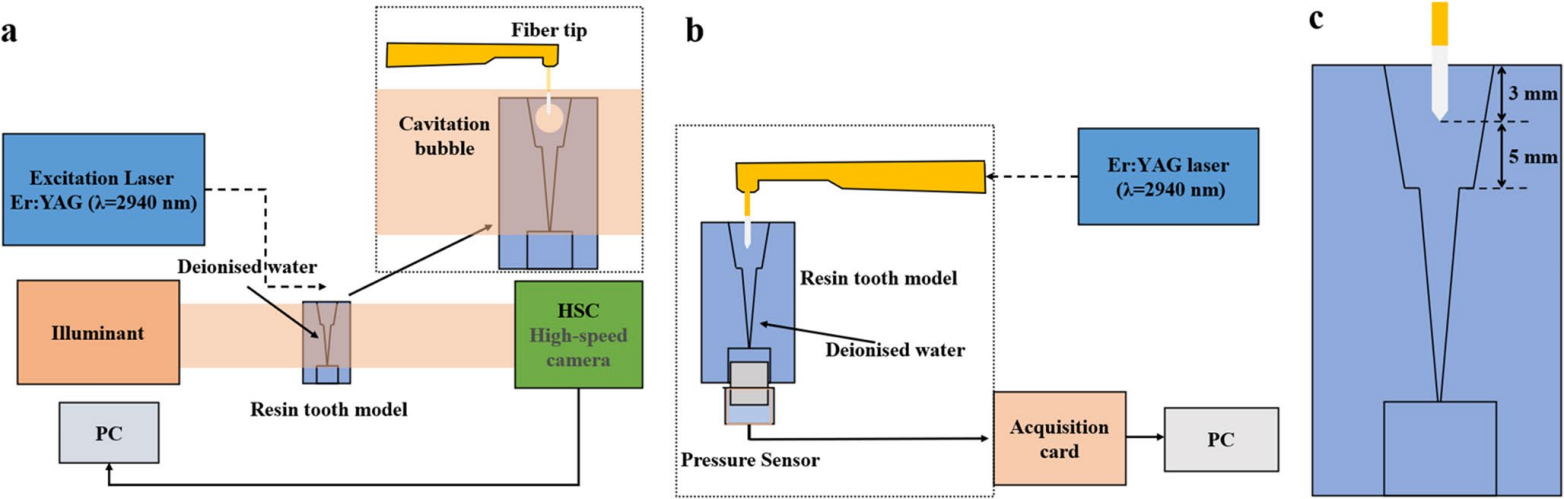
Schematic diagram of experimental working mode: (**a**) a vapor bubble dynamic observation and (**b**) TAPP measurement. (**c**) A schematic diagram of the insertion position of the laser tip

The Er: YAG laser device (Fotona, Slovenia) equipped with the auto-SWEEPS mode, with 20 mJ energy per pulse and 20 Hz frequency, was used (Fig. 2). A 600 μm diameter sapphire conical SWEEPS tip (model 89036, 9 mm length; Fotona, Slovenia) was attached to the H14 dental handpiece of an Er: YAG laser (LightWalker; Fotona, Slovenia). The tip was positioned 3 mm below the pulp chamber roof (or 5 mm above root canal orifice) using a micromanipulator, ensuring it remained centered and did not contact the canal walls (Fig. 2c). In the SWEEPS modality, the Er: YAG laser operates at a repetition frequency of 20 Hz, delivering 20 laser pulses per second. The ‘dual-pulse’ configuration refers to two consecutive pulses within this continuous pulse train. As illustrated in Fig. S1, each pair of consecutive pulses is separated by a fixed Tp, which is the key parameter varied in this study (120, 210, 300, 400, and 500 µs). Throughout the experiment, fresh deionized water will be continuously replenished to ensure that the root canal model is fully filled with deionized water and that the laser tip is always completely submerged in water. All experiments were conducted at controlled room temperature.

The optical setup is shown in Fig. 2a. First, the initial position of the laser working tip was calibrated using a micromeasurement positioning system, and a 75, 000 lm LED lighting system was set up on the dorsal side of the root canal model to ensure that the edge morphology of the bubbles in the liquid phase was clearly visible. Before the experiment, the root canal system was filled with deionized water. Subsequently, the Er: YAG laser was activated for irrigation, and a high-speed camera system (iSPEED726 mono144GB, iX Cameras Ltd, UK) with a frame rate of 2 × 10^5^ s^− 1^, shutter time of 5 µs, and resolution of 280 × 294 pixels was used to record changes in the dynamic morphology of bubbles within the pulp cavity under different Tp conditions.

The TAPP measurement system is shown in Fig. 2b. Before the experiment, the root canals were filled with deionized water. A stable baseline voltage was established by triggering the laser for 2 s. Subsequently, laser-activated irrigation was performed for 8 s. Throughout the experiment, fresh deionized water was continuously replenished to maintain liquid stability. The pressure signal at the root apex was converted into a voltage signal by a 50 kHz high-frequency pulsating pressure sensor and transmitted to the computer by a data acquisition card, which was ultimately used to calculate the TAPP values under different Tp conditions. Prior to measurements, the pressure sensor was calibrated in still deionized water under no-laser conditions for three 30 s runs (total 90 s) to establish a stable baseline voltage and confirm the absence of baseline drift. Calibration was accepted if the baseline voltage varied by ≤ 0.5% across the three runs.

### Image and data processing

#### Calculation of grayscale integral of secondary cavitation

**Fig. 3:**
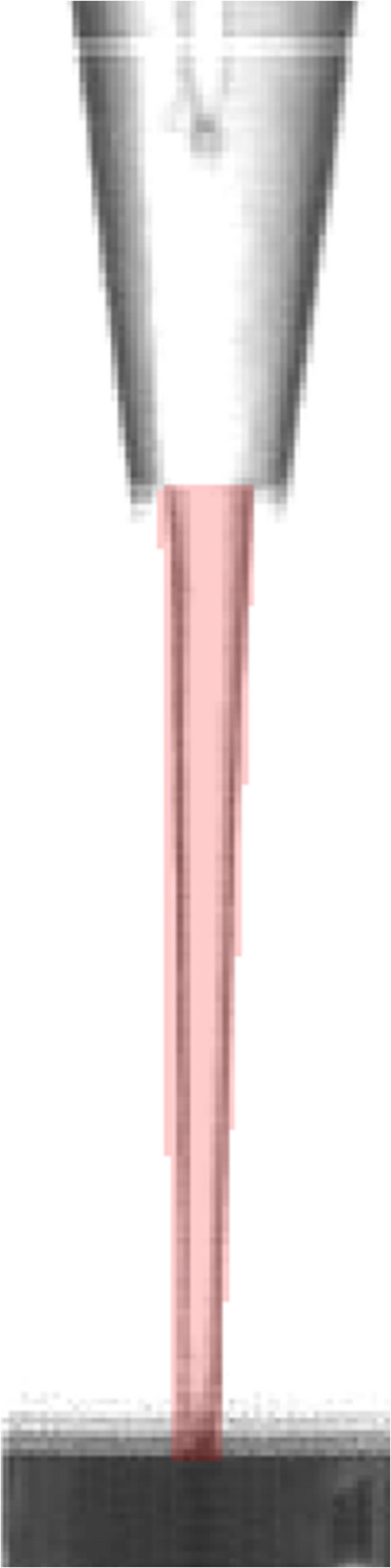
Schematic diagram of grayscale integration area of root canal. The red region represents the ROI used for pixel intensity measurement

With the image captured by the high-speed camera before laser activation as the background, the MATLAB software was used to mark a region of interest (ROI) corresponding to the entire root canal lumen—from the root canal orfice to the apical foramen—as shown in the red region in Fig. 3. Within this ROI, the grayscale value (pixel intensity) of each pixel was measured. Subsequently, the grayscale integral was calculated by summing the grayscale values of all pixels within the ROI across every frame of the high-speed image sequence captured during the entire bubble’s oscillation period (Tosc). This grayscale integral serves as a surrogate for the relative volume of secondary cavitation integrated over time within the restricted root canal space.

#### Calculation of TAPP

Voltage data collected from the root-tip area for 10 s were converted into relative pressure values using the formula $\:P=(U-{U}_{0})\bullet\:r$, where P is the relative pressure value, $\:\mathrm{U}$ is the real-time voltage, $\:{\mathrm{U}}_{0}$ is the baseline voltage, and $\:\mathrm{r}$ is the conversion rate constant ($\:\mathrm{r}$ = 1.73 × 10^4^ Pa/V for the sensor; XCS-190SM-30 A, Kulite Semiconductor Products Inc, America)). The maximum relative positive pressure value under different Tp conditions is referred to as the maximum positive pressure. To avoid the numerical fluctuations that occur in the initial stage of laser activation, TAPP is calculated based on the arithmetic mean of the maximum positive pressure values of the last 40 pulses, and during this period, the pressure signal remains stable.

#### Calculation of maximum diameter and volume of bubbles

Maximum bubble volume (V_max_) was calculated using MATLAB by measuring the longitudinal (d_1_) and transverse (d_2_) diameters of the bubble at its maximum expansion (Fig. S2). The formula is: V_max_ = 4/3 × π × (d_1_/2) × (d_2_/2)^2^. The pulp chamber opening is used as a 4 mm calibration baseline.

## Statistical analysis

This study used GraphPad 9.0, to perform one-way ANOVA on root canal grayscale integration, and TAPP, with *P* < 0.05 indicating statistical significance. All data are expressed as mean ± standard deviation (SD).

## Results

### Image features of vapor bubbles formed by SWEEPS dual-pulse mode

**Fig. 4:**
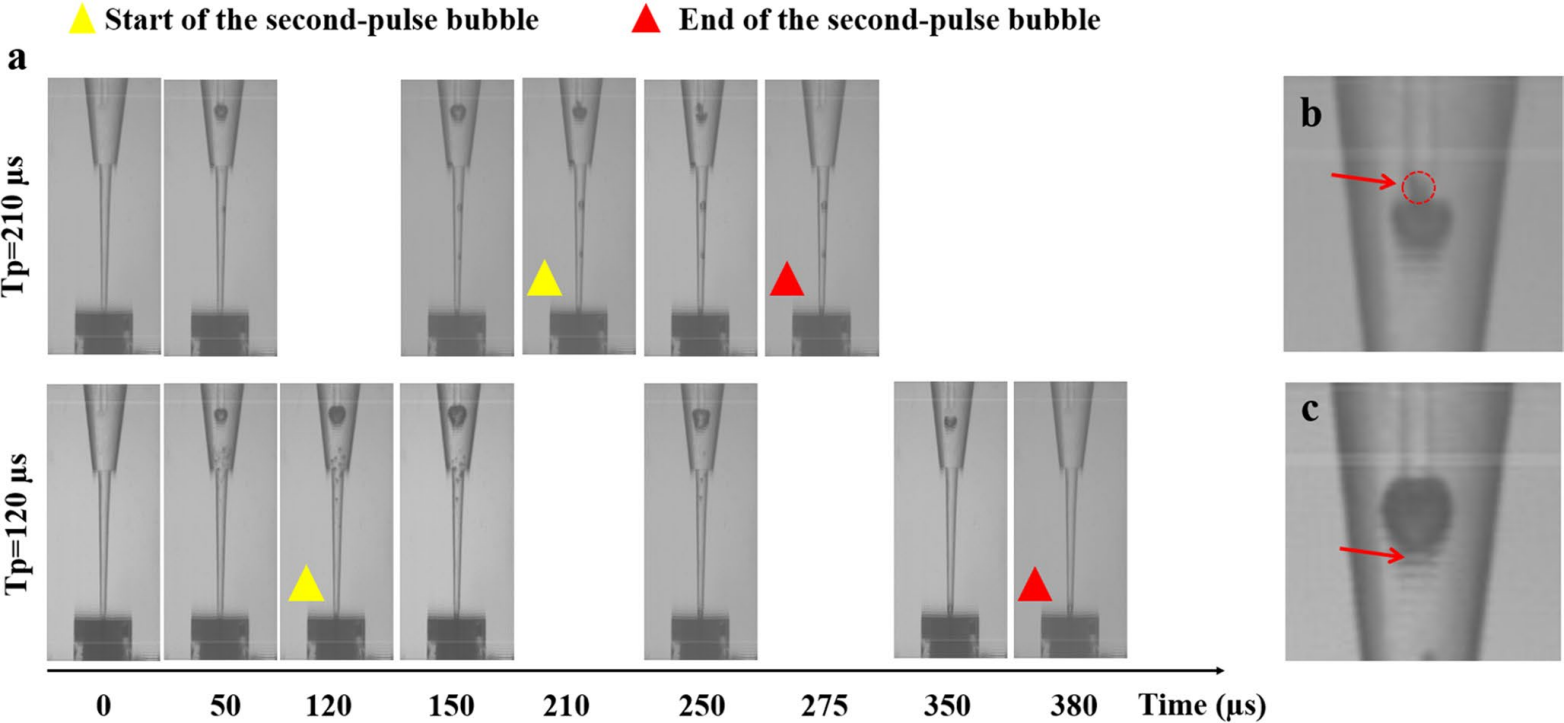
Typical sequence of acquired images during laser bubble generation. (**a**) Relationship between double pulse bubbles at Tp of 120 and 210 µs. Local magnified view of the moment when the second-pulse bubble was formed at Tp = 210 (**b**) and 120 (c) µs

The snapshots show that when Tp was 120 and 210 µs, the vapor bubbles generated by the second pulse merged with those generated by the first pulse, forming larger bubbles (Fig. 4). Moreover, when Tp was 120 µs, the maximum volume of the merged bubble was larger than that when Tp was 210 µs (Table S1).

**Fig. 5:**
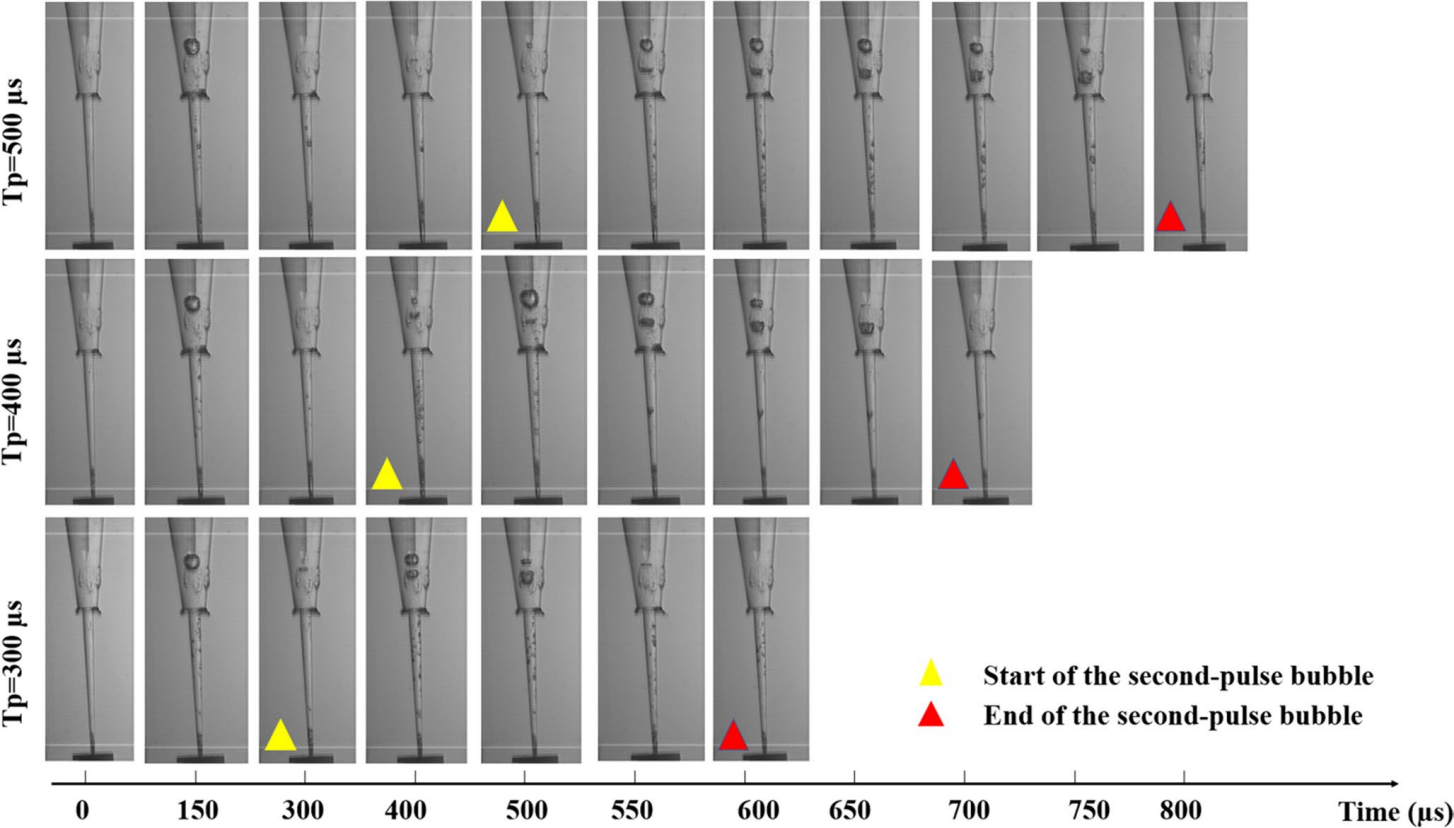
Relationship between double pulse bubbles at Tp of 300, 400, and 500 µs

Furthermore, when Tp was 300, 400, and 500 µs, the second pulse can generate two separate vapor bubbles, thereby forming a relatively independent double bubble structure (Fig. 5). Simultaneously, the vapor bubbles generated by the second pulse impacted the vapor bubbles of the first pulse.

### Effects of Tp on root canal grayscale integrations

**Fig. 6:**
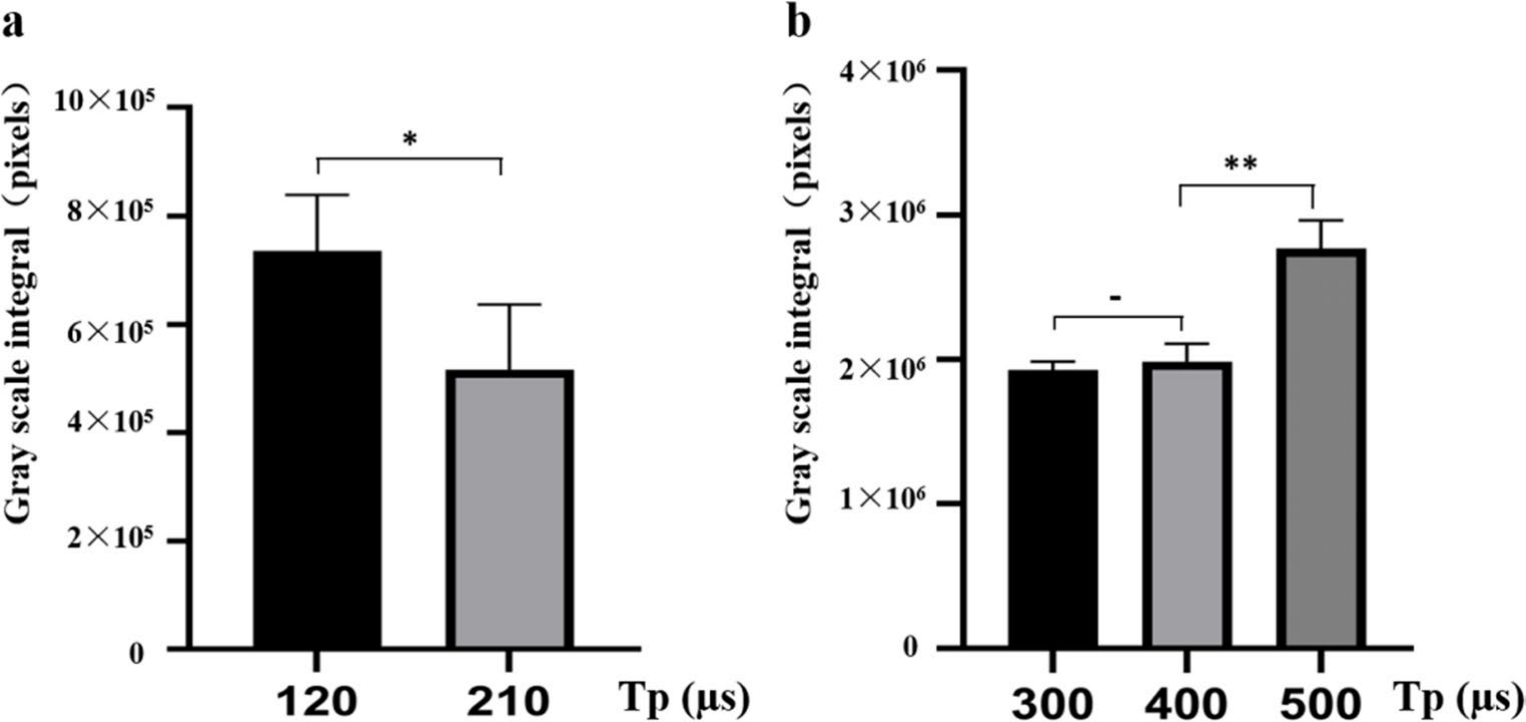
Grayscale integrals of secondary cavitation activated by Er: YAG laser for irrigation under different Tp conditions: (a) 120 and 210 µs and (b) 300, 400, and 500 µs. -: No statistical difference. *: *p*<0.05. **: *p*<0.01

When Tp was 120 µs, the grayscale integral of secondary cavitation was significantly higher than when Tp was 210 µs, and the difference was statistically significant (P ˂ 0.05), as shown in Fig. 6a; Table 1. When Tp was 500 µs, the grayscale integral of secondary cavitation was significantly higher than the corresponding values when Tp was 300 and 400 µs (P ˂ 0.05). When Tp was between 300 and 400 µs, there was no statistically significant difference in the grayscale integral of secondary cavitation (*P* > 0.05), as shown in Fig. 6b; Table 1.

**Table 1:** Grayscale integral and TAPP results for different Tp values

Group	Tp (µs)				
	120	210	300	400	500
Grayscale integral (× 10^6^ pixel)	0.074 ± 0.010	0.052 ± 0.012^a^	1.926 ± 0.06^a, b^	1.986 ± 0.135^a, b^	2.767 ± 0.195^a, b,c, d^
TAPP (× 10^3^ Pa)	3.520 ± 0.291	2.835 ± 0.047^a^	2.807 ± 0.099^a^	3.128 ± 0.182^a, c^	3.398 ± 0.068^b, c,d^

^a^Compared with 120 µs within the group, *P* < 0.05

^b^Compared with 210 µs within the group, *P* < 0.05

^c^Compared with 300 µs within the group, *P* < 0.05

^d^Compared with 400 µs within the group, *P* < 0.05

### Effects of Tp on TAPP

**Fig. 7:**
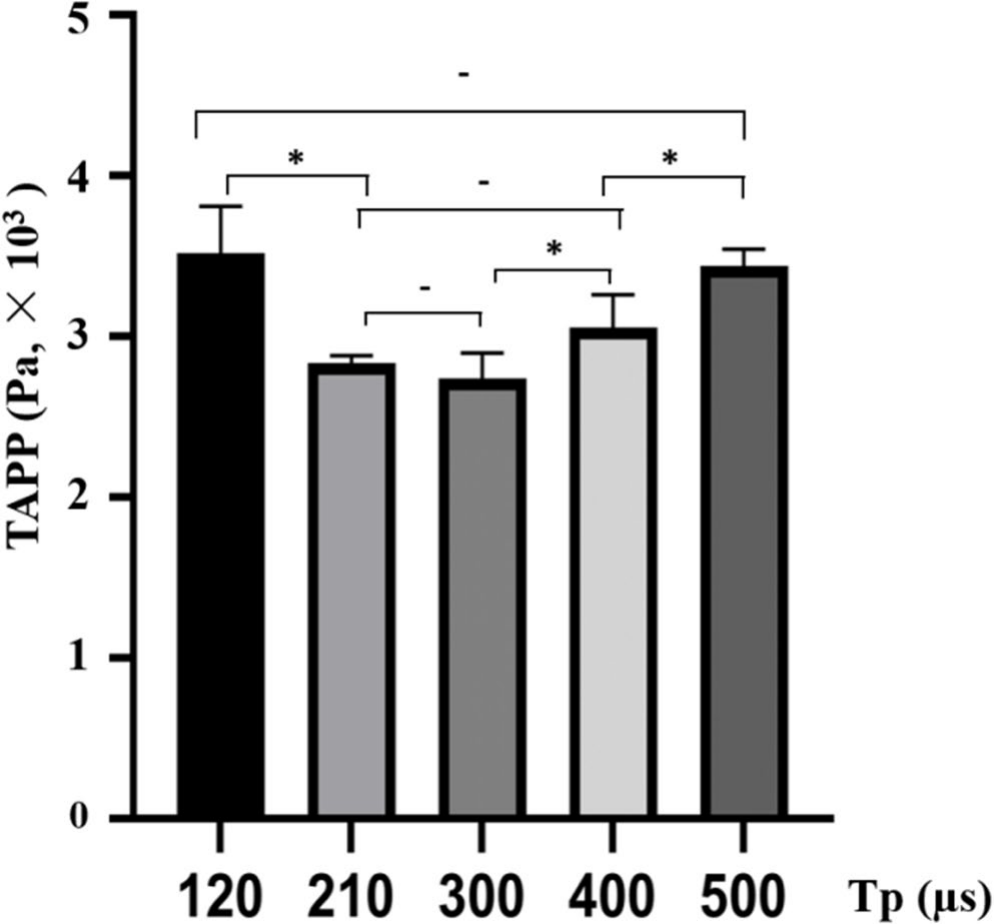
Relationship between Tp and TAPP. -: No statistical difference. *: *p*<0.05. **: *p*<0.01

The TAPP values measured during Er: YAG laser-activated irrigation under different Tp conditions are shown in Fig. 7; Table 1. When Tp was 120 µs, the TAPP value was significantly higher than that when Tp was 210 µs (*P* < 0.05). Similarly, when Tp was 500 µs, the TAPP value was significantly higher than the corresponding values when Tp was 300 and 400 µs (*P* < 0.05). However, no significant differences were observed in TAPP for Tp ranges of 120–500 µs and 210–300 µs (*P* > 0.05).

## Discussion

The aim of this study is to investigate the effect of different pulse interval times (Tp) on the fluid dynamics of SWEEPS dual pulse Er: YAG laser irrigation. The null hypothesis—that no significant differences exist in bubble volume or TAPP among Tp values of 120, 210, 300, 400, and 500 µs—is rejected. Therefore, the alternative hypothesis is accepted: different Tp settings do produce significantly different fluid dynamics.

Gregorčič et al. [17] discovered in 2014 that the dual pulse laser mode can generate resonance effects. They pointed out that when the second laser pulse is delivered at the end or later of the collapse of the first bubble, it can significantly enhance the mechanical energy of the secondary oscillation, which is called the resonance effect. The TP time nodes designed in this article are considered to be 120, 210 µs, and 300, 400, and 500 µs, where 120, 210, and 300 µs take into account the period of the first bubble (Figs. S3 and S4). Our core finding is that when the second laser pulse is introduced during the bubble expansion stage induced by the first pulse (i.e. before the bubble reaches its maximum volume), it can significantly enhance the final volume of the first bubble, similar to previous research results, and may also be due to the “resonance effect”. Lukač and Jezeršek’s study in 2018 also confirmed that the growth pressure generated by a properly synchronized second laser pulse can accelerate the collapse of the first bubble, thereby generating shock waves in a restricted space [18] which is consistent with the trend of our experimental results.

The TP time nodes of 300, 400, and 500 µs designed in this article mainly consider the position of the first bubble from the laser tip when the second pulse is output. The TP time nodes of 300, 400, and 500 µs are designed to observe the position of the first bubble from the laser tip during the second pulse output stage. The study by Xinyu He et al. [16] in free water also showed that different Tps significantly affect the interaction mode of dual pulse bubbles. When the first bubble reaches a certain distance from the laser tip, inputting the second pulse can achieve an enhancement effect. We further consider extending this principle to a more clinically relevant root canal model - the restricted water model.

In addition, the enhanced fluid dynamics effects observed in this study provide physical support for the advantages of SWEEPS technology in terms of rinse efficiency. For example, the study by De Meyer et al. [19] showed that laser activated rinsing (LAI) is superior to ultrasonic rinsing (UAI) in clearing biofilms, especially when using physiological saline as the rinsing agent. This improvement in physical clearance ability may be due to the strong fluid motion generated by LAI.

Although this study provides important experimental evidence, there are still some limitations that need to be addressed in future research.

Firstly, this experiment used a transparent resin root canal model instead of real teeth. Compared with natural teeth, resin models have differences in material properties such as thermal conductivity and surface roughness, as well as anatomical structures of the pulp cavity such as size, taper, and curvature. As Lukač et al. [18] pointed out, the oscillation period of bubbles strongly depends on the size of the cavity; In smaller cavities, the oscillation period will be significantly prolonged. Therefore, the conclusion of “inject during the bubble expansion stage” determined in this study may have significant differences in the optimal Tp value among teeth with different pulp cavity volumes. In addition, for the convenience of high-speed camera observation, we adopted a single straight root canal model, which simplifies the complex fluid dynamics environment of multiple, curved root canals commonly seen in clinical practice. At the same time, there are some non physiological structures in the model (such as the “step” structure at the pulp floor) that do not exist in real teeth and may affect local fluid motion patterns.

Secondly, this study only evaluated the hydrodynamic effects through high-speed photography and peak pressure measurement, lacking direct validation of the biological efficacy of debris removal or biofilm removal. Many scholars have made outstanding contributions in this area. For example, DiVito et al. [20] demonstrated that PIPS technology can effectively remove smearing layers at low energy settings, while De Moor et al. [21] confirmed that laser activated rinsing is more effective than traditional syringe rinsing in cleaning debris in irregular areas of root canals. In addition, in a study conducted by De Meyer et al. [19], a dual strain biofilm model was used to quantitatively demonstrate that LAI technology using Er: YAG laser can reduce bacterial numbers by more than 4 logarithmic orders of magnitude when processing 40 mJ pulse energy, which is far superior to ultrasonic oscillation washing. Our fluid dynamics results provide a reasonable physical mechanism explanation for these biological effects to some extent.

Finally, only deionized water was used as the flushing solution in the experiment, and sodium hypochlorite or ethylenediaminetetraacetic acid solutions widely used in clinical practice were not employed. Different rinsing solutions may have different absorption coefficients for Er: YAG laser (2.94 μm wavelength), affecting the dynamic behavior of bubbles [22]. For example, the absorption characteristics of sodium hypochlorite solution may differ from those of water, resulting in the generation of bubbles with different volumes and oscillation periods [21]. In addition, the synergistic effect between the chemical activity of the flushing solution (such as the bactericidal and tissue dissolving ability of sodium hypochlorite) and physical fluid dynamics is the key factor determining the final clinical efficacy, and the design of this study cannot evaluate this synergistic effect.

## Conclusion

In this in vitro resin root canal model with deionized water, SWEEPS dual-pulse Er: YAG laser-activated irrigation with Tp = 120 or 500 µs produced greater secondary cavitation volume and higher apical pressure than intermediate Tp values (210–400 µs). As a parameter of Er: YAG laser-activated irrigation, Tp has the potential to influence the irrigation effect in this experimental setting.

## Supplementary Information

Supplementary Material 1.

## Data Availability

Raw data for this study can be obtained upon request from the corresponding author.

## Abbreviations

SWEEPS: Shock wave-enhanced emission photoacoustic streaming
Er:YAG: Erbium-doped yttrium aluminum garnet
Tp: Temporal delay
TAPP: Transient peak pressure
SD: Standard deviation
ROI: Region of interest
Tosc: Bubble’s oscillation period
PIPS: Photon-induced photoacoustic streaming
SNI: Syringe needle irrigation
PUI: Passive ultrasonic irrigation
LAI: Laser activated rinsing
UAI: Superior to ultrasonic rinsing
