# Supplementary material for: Influence of temporal delay in SWEEPS dual-pulse Er: YAG laser-activated irrigation on fluid dynamics in confined domain environment: an in vitro study
Source: Lasers Med Sci. 2026 Jul 22;41(1):157. doi: 10.1007/s10103-026-04947-9 (PMC13388415; doi:10.1007/s10103-026-04947-9)
Supplement: Supplementary file 1 — Supplementary Material 1. [file 10103_2026_4947_MOESM1_ESM.docx]

**Supplementary Information**

**Influence of temporal delay in SWEEPS dual-pulse Er:YAG laser-activated irrigation on fluid dynamics in confined domain environment: An in vitro study**


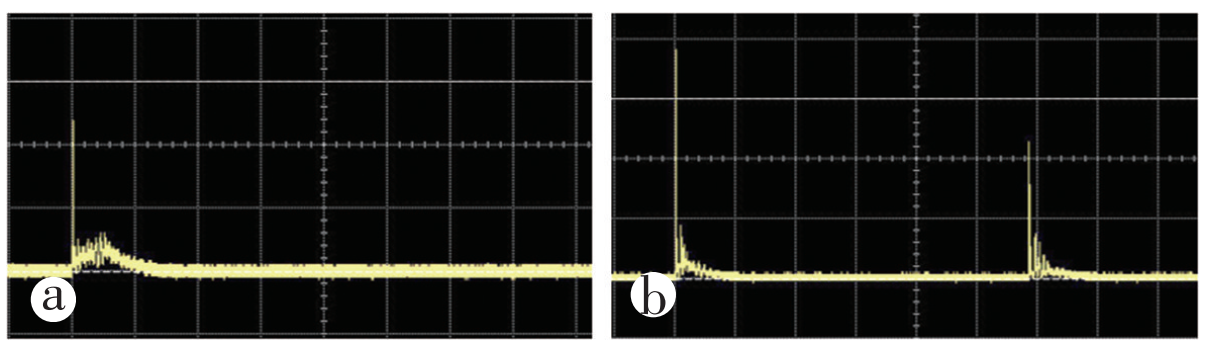


**Fig. S1** Pulse waveforms of Er: YAG laser in different working modes. (a) PIPS technology pulse waveform diagram, (b) SWEEPS technology pulse waveform diagram, with the horizontal axis in each grid representing 50 μs


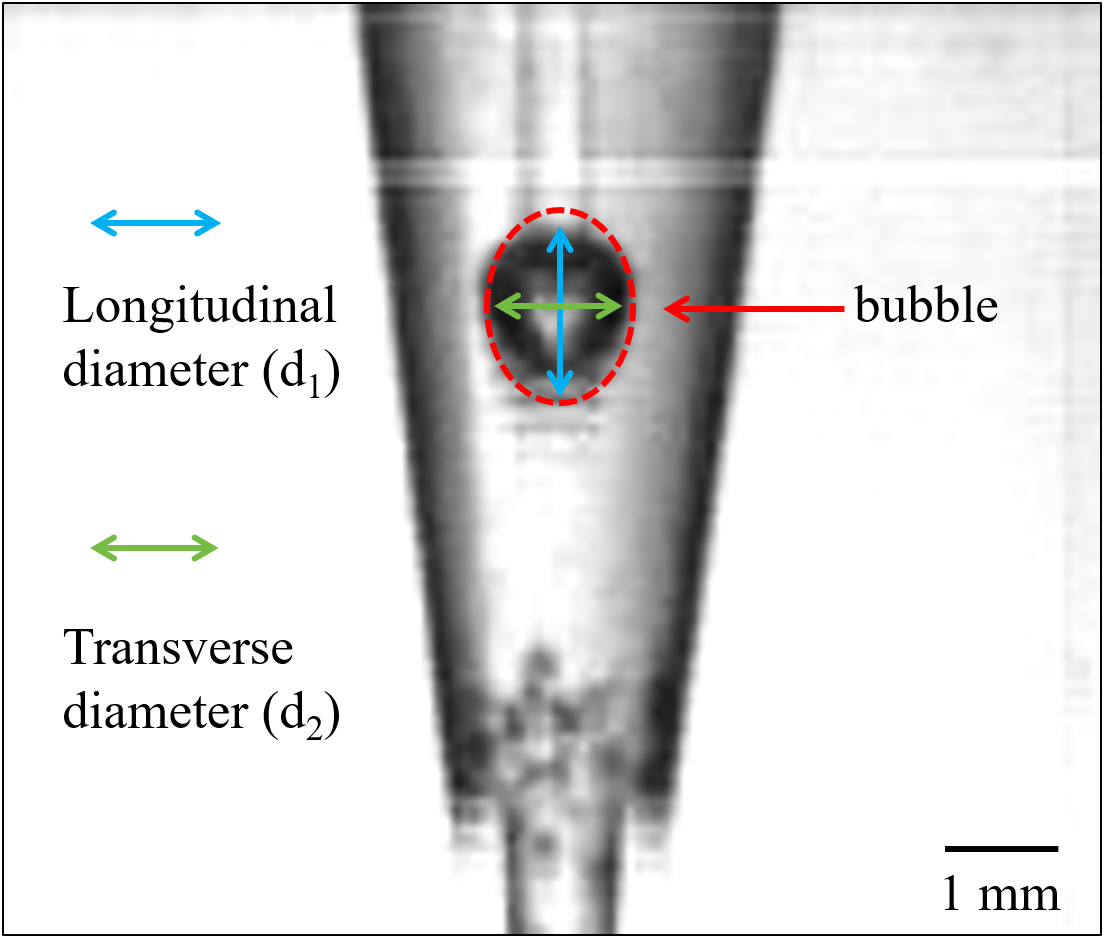


**Fig. S2** Measurement of the maximum diameter of bubbles

**Table S1** Comparison of maximum bubble volume between the 120 and 210 μs groups

| Group | n | V_max_ (mm^3^) | Mean difference (mm^3^) | 95% CI (mm^3^) | t | df | P value |
| --- | --- | --- | --- | --- | --- | --- | --- |
| 120 μs | 4 | 2.119 ± 0.070 | 1.091 | 0.825-1.356 | 13.075 | 3 | 0.001 |
| 210 μs | 4 | 1.029 ± 0.104 |  |  |  |  |  |

**Regarding the selection of Tp**

During the Er:YAG laser-activated root canal cleaning process, vapor bubbles are generated due to cavitation effects. When a single pulse energy is input, two expansion-collapse processes of the induced vapor bubble volume can be observed using a high-speed camera (Figs. S3 and S4). The first bubble oscillation (a - g in Figure S3; i - iii in Figure S4) includes the growth of bubble to its collapse. Similarly, the second bubble oscillation (g to l in Figure S3; iii to v in Figure S4) includes the growth of bubble to its collapse.

Therefore, based on the two expansion-collapse oscillation patterns of bubble volume, Tp was selected as 120, 210, 300, 400, and 500 µs, to study all the dynamic stages of vapor bubble evolution, namely, the start of the first pulse vapor bubble volume, the first expansion stage, the first collapse stage, the collapse to the minimum value, the second expansion stage, and the second collapse stage.


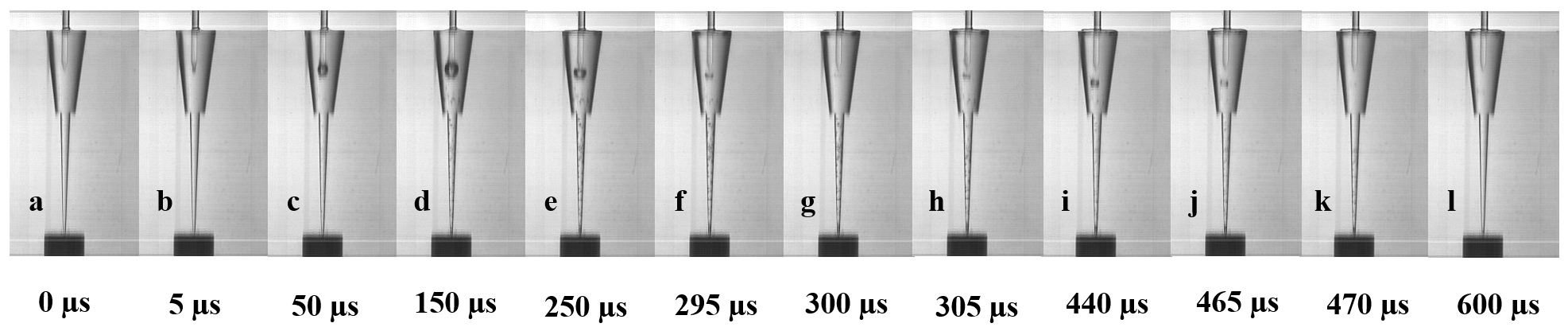


**Fig. S3** Evolution process of typical vapor bubbles in single pulse energy activated oscillation washing. First expansion and collapse stages of vapor bubbles: (a)–(d) vapor bubble expansion stage, (d) time when the vapor bubble volume reaches its maximum, and (d)–(g) vapor bubble collapse stage; second expansion and collapse stages of the vapor bubbles: (g)–(i) vapor bubble expansion stage, (i) the time at which the vapor bubble volume reaches its maximum, and (i)–(l) vapor bubble collapse stage


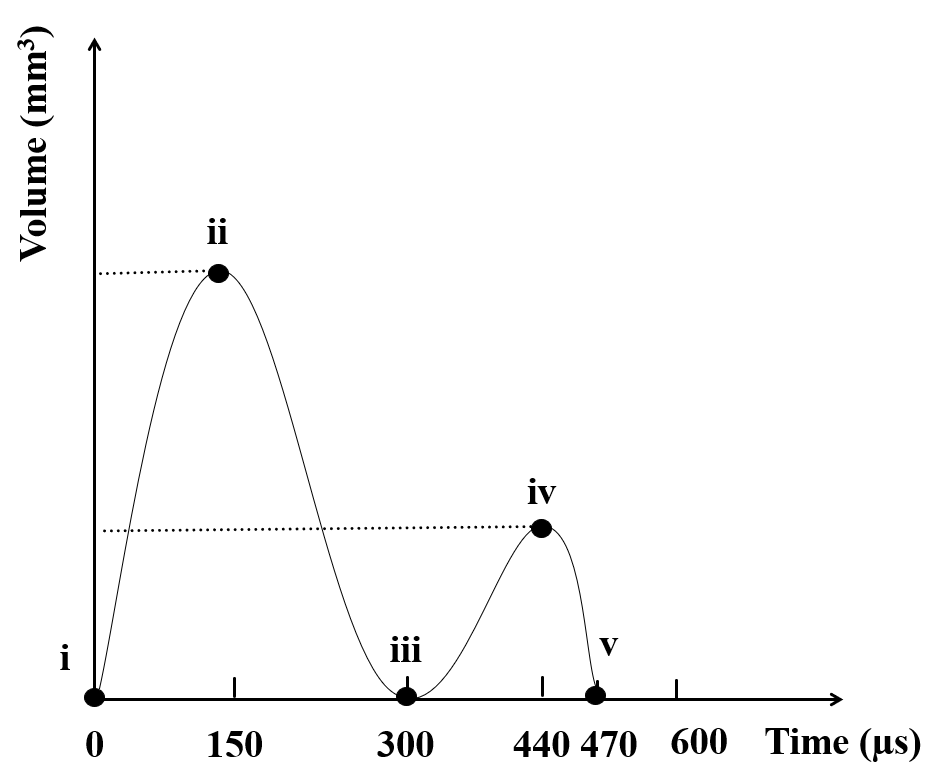


**Fig. S4** Volume curve of vapor bubbles during single pulse energy activated irrigation: first expansion stage of vapor bubbles (ⅰ–ⅱ); first collapse stage of vapor bubbles (ⅱ–ⅲ); second expansion stage of vapor bubbles (ⅲ–ⅳ); and second collapse stage of vapor bubbles (ⅳ–ⅴ)

**The effect of different Tp on fluid dynamics**

This study used the SWEEPS technique to set five different Tp values (120, 210, 300, 400, and 500 µs). When Tp = 210 and 300 µs, there was no statistical difference in TAPP, and both were smaller than the TAPP at Tp = 120 µs. This indicates that when the second pulse of energy was injected during the expansion stage of the first pulse of the vapor bubbles, the resulting fluid dynamics were stronger than when energy was injected during the collapse stage. This might be due to the fact that energy injection during the expansion stage effectively increased the maximum volume of the first bubble, thereby enhancing the hydrodynamic force of the first pulse. When Tp = 300, 400, and 500 µs, there was interaction between the second pulse vapor bubble and the first pulse vapor bubble, and TAPP, root canal grayscale integration value, and double bubble interaction distance increased with the increase of Tp. This suggests that during the second expansion and collapse stage of the first-pulse vapor bubble, the closer the second pulse energy is injected towards the end of the second collapse, the stronger the interaction distance between the two bubbles and the stronger the fluid dynamics. This also suggests that in clinical practice, fluid dynamics during root canal flushing can be effectively enhanced without increasing the maximum volume of vapor bubbles, which can effectively overcome the spatial limitation of vapor bubble expansion caused by the fixed pulp chamber structure activated by single-pulse energy during flushing.

It is worth noting that although there is no significant difference in TAPP between Tp of 120 and 500 µs (P > 0.05), indicating similar effects in improving fluid dynamics, the bubble period corresponding to Tp of 120 µs is 380 µs, whereas the bubble period at Tp of 500 µs is 800 µs, indicating a significant difference in bubble period between the two. This indicates that under similar fluid dynamics, a Tp of 120 µs has a higher flushing efficiency.
